# Concentrated Growth Factor Matrices Prepared Using Silica-Coated Plastic Tubes Are Distinguishable From Those Prepared Using Glass Tubes in Platelet Distribution: Application of a Novel Near-Infrared Imaging-Based, Quantitative Technique

**DOI:** 10.3389/fbioe.2020.00600

**Published:** 2020-06-16

**Authors:** Sadahiro Yamaguchi, Hachidai Aizawa, Atsushi Sato, Tetsuhiro Tsujino, Kazushige Isobe, Yutaka Kitamura, Taisuke Watanabe, Hajime Okudera, Carlos Fernando Mourão, Tomoyuki Kawase

**Affiliations:** ^1^Tokyo Plastic Dental Society, Tokyo, Japan; ^2^Department of Oral Surgery, Dentistry School, Fluminense Federal University, Rio de Janeiro, Brazil; ^3^Division of Oral Bioengineering, Institute of Medicine and Dentistry, Niigata University, Niigata, Japan

**Keywords:** concentrated growth factors, platelet, distribution, near-infrared imaging, CD41

## Abstract

Platelet-rich fibrin (PRF) matrices were originally prepared using plain glass tubes without the aid of coagulation factors because coagulation factor XII is activated by glass surfaces. Recently, the use of silica-coated plastic tubes as a substitute of glass tubes has been recommended for PRF preparation. This recommendation is owing not only to the shortage of glass tubes for medical use in the market, but also the higher coagulation activity of silica-coated plastic tubes and equal quality of PRF. However, these matrices are not the same. To evaluate the differences, we compared glass- and silica-coated plastic tubes in terms of platelet distribution and quantity in concentrated growth factors (CGF). CGF matrices were immediately prepared from freshly collected blood samples, fixed after red thrombus removal, and divided into two equal pieces sagittally. One piece was used for CD41 detection and the other was applied as an isotype control. Platelet distribution in CGF matrices was examined, without embedding or sectioning, by a novel method using invisible near-infrared imaging. The dehydrated membranous CGF matrix was more transparent. Thus, the fluorescence signal was clearly detectable with less scattering. Platelets were distributed mainly in the distal side of the glass-prepared CGF matrix, but homogeneously in the silica-prepared CGF matrix. Platelet count was positively correlated with fluorescence intensity. Although not yet fully developed, this imaging technique enabled us to recognize the differences in platelet distribution and quantity in CGF matrices by excluding bias caused by the technical limitations of scanning electron microscopy and conventional immunohistochemical methods.

## Introduction

Almost three decades have passed since the pathophysiological roles of platelets in wound healing have attracted attention and platelets have been applied in the form of platelet-rich plasma (PRP) in regenerative therapy (Marx et al., 1998). In the meantime, various types of platelet concentrates have been developed (Kawase, 2015). Because of their simple operation without specific technical requirements, platelet-concentrated fibrin matrices, generally designated as platelet-rich fibrin (PRF) (Kawase and Tanaka, 2017), have been recently used preferentially in regenerative dentistry. PRF preparation is triggered simply through the activation of intrinsic coagulation pathways by the inner surface of glass tubes without the aid of any coagulation factor during or after blood collection. However, from a biomedical point of view, platelets are entrapped in insoluble fibrin matrices and therefore can hardly be quantified for quality assurance of individual PRF preparations and subsequent PRF therapy.

Platelets contained in fibrin matrices have been conventionally evaluated by the subtraction method (Watanabe et al., 2017). We pointed out the inaccuracy of this indirect method and developed a direct method using tissue-plasminogen activator to efficiently digest fibrin matrices without severely damaging the platelets (Kitamura et al., 2018b). This method is superior in quantification, recovery rate, and reproducibility. However, to evaluate platelet distribution in fibrin matrices, histological and microscopic methods are required. In earlier studies, platelet distribution was examined using scanning electron microscopy (SEM), which is powerful for the observation of platelets distributed on the surface of fibrin matrices. However, it can hardly be used to examine platelet distribution on the inside of the matrix, and platelets were identified simply by their morphology. In contrast, immunohistochemical methods can clearly and specifically demonstrate platelet distribution in thin sections. Although it is possible to reconstruct three-dimensional images of the original specimens from accumulated section images, it is inferior in quantification.

In this study, we focused on the high transmittance of near-infrared (NIR) light in our body (Smith et al., 2009) and developed a novel method to quantitatively visualize platelet distribution in insoluble platelet-concentrated fibrin matrices, without paraffin embedding or sectioning. Owing to technical and interpretation biases, it is generally difficult to appropriately reconstruct a three-dimensional platelet distribution by the conventional histochemical examination. Light in the visible range (400–660 nm), which is used for conventional immunofluorescence staining, cannot efficiently pass through biological materials. We applied the NIR imaging technique to examine the effects of glass and silica-coated plastic tubes on platelet distribution in concentrated growth factors (CGF), a type of PRF matrix.

## Materials and Methods

### Preparation of CGF Matrices

Blood samples were collected from 15 healthy non-smoking male and female volunteers aged 31–72 years. Depending on the purpose of each experiment, minimal but essential numbers of donors were randomly selected for sample collection. In the experiments using CGF matrices prepared from whole-blood samples (see **Figure 7**), blood samples were collected twice with intervals of 1 week at minimum. The numbers of samples used are stated in individual figure legends. The study design and consent forms for all procedures performed were approved by the ethics committee for human participants of the Niigata University School of Medicine (Niigata, Japan) in accordance with the Helsinki Declaration of 1964 as revised in 2013.

Blood (~9 mL) was collected from the volunteers by peripheral venipuncture in one of the antecubital fossa veins into plastic vacuum plain blood collection tubes (Neotube #NP-PN0909; NIPRO, Osaka, Japan). The blood sample was immediately transferred to glass tubes (NICHIDENRIKA-GLASS Co., Ltd., Kobe, Japan) or silica-coated plastic tubes (Neotube #NP-PS0909; NIPRO) and placed on a fixed-angle rotor (Medifuge centrifuge, SILFRADENT, Santa Sofia, Italy) to start the centrifugation process (Kobayashi et al., 2012; Tsukioka et al., 2019). Especially in the case of silica-coated tubes, coagulation began as soon as blood collection started and therefore, fibrin clot formation varied with the order of blood collection. To minimize such variations, we first collected blood samples in plain plastic tubes and then transferred the samples into glass- or silica-coated tubes (eight tubes maximum) within a short period of time (~2 min).

There could be an additional option, i.e., use of silica-coated or silica-containing glass tubes, to more clearly highlight the effects of silica microparticles on CGF matrix formation. From a basic scientific point of view, this option was interesting enough to stimulate our curiosity. However, we did not expand this pursuit for the following 2 reasons. First, because both glass surface and silica microparticle are similarly capable of activating coagulation cascade, an additive or synergistic effect of these materials could occur on fibrin clot formation. If so, it may be difficult to identify coagulation induced predominantly by silica microparticles alone. Second, it is regarding to the commercial and clinical availability. Either silica-coated or silica-containing glass tubes are not commercially available or not used in clinical settings. We may use home-made silica-containing glass tubes; however, we are concerned that readers may be confused about our experimental design or strategy. To avoid unexpected outcomes, we decided not to perform such an additional experiment.

After cutting off red blood cell (RBC) fraction, i.e., clot, using scissors, CGF matrices were gently washed with phosphate-buffered saline (PBS) and fixed in 10% neutralized formalin (FUJIFILM Wako Pure Chemical Corp., Osaka, Japan) for 3–5 h in plastic tubes to prevent the collapse of CGF shape. Thereafter, CGF matrices were transferred to PBS and stored at 4°C until use, usually within 48 h.

To obtain the basic characteristics of individual blood samples, the number of platelets and other blood cells in whole-blood samples and PRP preparations was determined using an automated hematology analyzer (pocH 100iV, Sysmex, Kobe, Japan).

### Modified Immunohistochemical Visualization of Platelet Distribution in CGF Matrices

**Figure 1:**
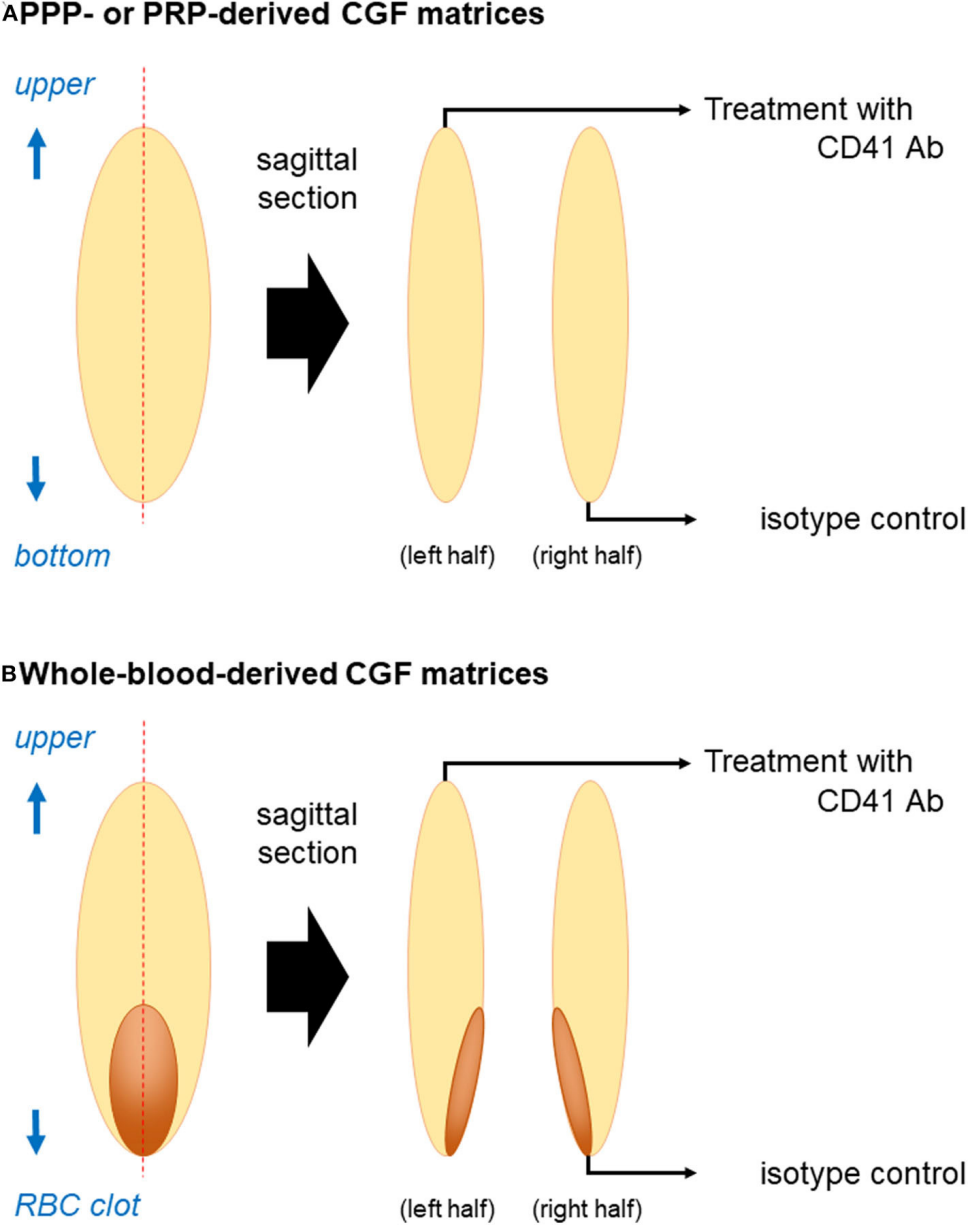
Illustration of sample preparation for immunohistochemical examination. **(A)** PPP- or PRP-derived fibrin matrices and **(B)** whole-blood-derived CGF matrices were divided into two pieces using a rotary cutter. The cross-sectional area was unfolded if the edge (surface of the fibrin matrix) was folded to be maximized and placed on the lid of a culture dish before dehydration on KimWipes. Note: “RBC clot” is equal to “red thrombus”.

Fixed CGF matrices were equally divided into two pieces. One piece was used for the detection of CD41^+^ platelets and the other was used as an isotype control (Figure 1). The CGF pieces were dehydrated using KimWipes (Kimberly-Clark Corp., Dallas, TX, USA) and washed twice in PBS containing 0.1% Tween 20 (0.1T-PBS) in 6-well plates for 10 min using a vortex mixer. After dehydration, the CGF pieces were blocked for 24 h in 2% Block Ace solution dissolved in 0.1T-PBS (BA-TPBS) in 2-mL sample tubes at 4°C.

The CGF pieces were then dehydrated, washed twice as described above, and probed with a mouse monoclonal human CD41 antibody (BioLegend, San Diego, CA, USA) at 3:1000 dilution in a 1:1 mixture of BA-TPBS and IMMUNOSHOT mild (Cosmobio, Tokyo, Japan) for 24 h at 4°C. The CGF isotype controls were treated with a non-immunized mouse IgG (BioLegend).

After primary antibody treatment, the CGF pieces were dehydrated, washed four times in 0.1T-PBS using a vortex mixer, and probed with NIR dye-conjugated goat anti-mouse IgG (1:4,500 dilution in BA) (iFluor 790; AAT Bioquest, Inc., Sunnyvale, CA, USA) for 90 min at 4°C.

The CGF pieces were dehydrated and washed four times with 0.1T-PBS using a vortex mixer. After removing excess buffer solution, wet CGF pieces were placed on the lids of 100-mm culture dishes and scanned at 800 nm using a Pearl NIR imaging system (Li-Cor, Lincoln, NE, USA). Then the CGF pieces were dehydrated using KimWipes and scanned again. This imaging system can detect two wavelengths (700 and 800 nm). We confirmed in a preliminary study that the longer wavelength produced significantly higher transmission. Thus, presently we used iFluor 790 dye. In this imaging system, the dye is excited by an 800 nm channel laser source (solid-state laser diode at 785 nm) and an emission at 820 nm is detected using a cooled CCD detector.

The total florescence intensity of each half of the CGF matrix or membrane was measured using software provided by Li-Cor and the fluorescence intensity of the background was subtracted from that of the matrix. The fluorescence intensity of CD41 antibody-reactive proteins was expressed by the ratio of CD41 to the corresponding isotype control.

### Correlation Between Fluorescence Intensity and Fluorescence Dye Concentration

**Figure 2:**
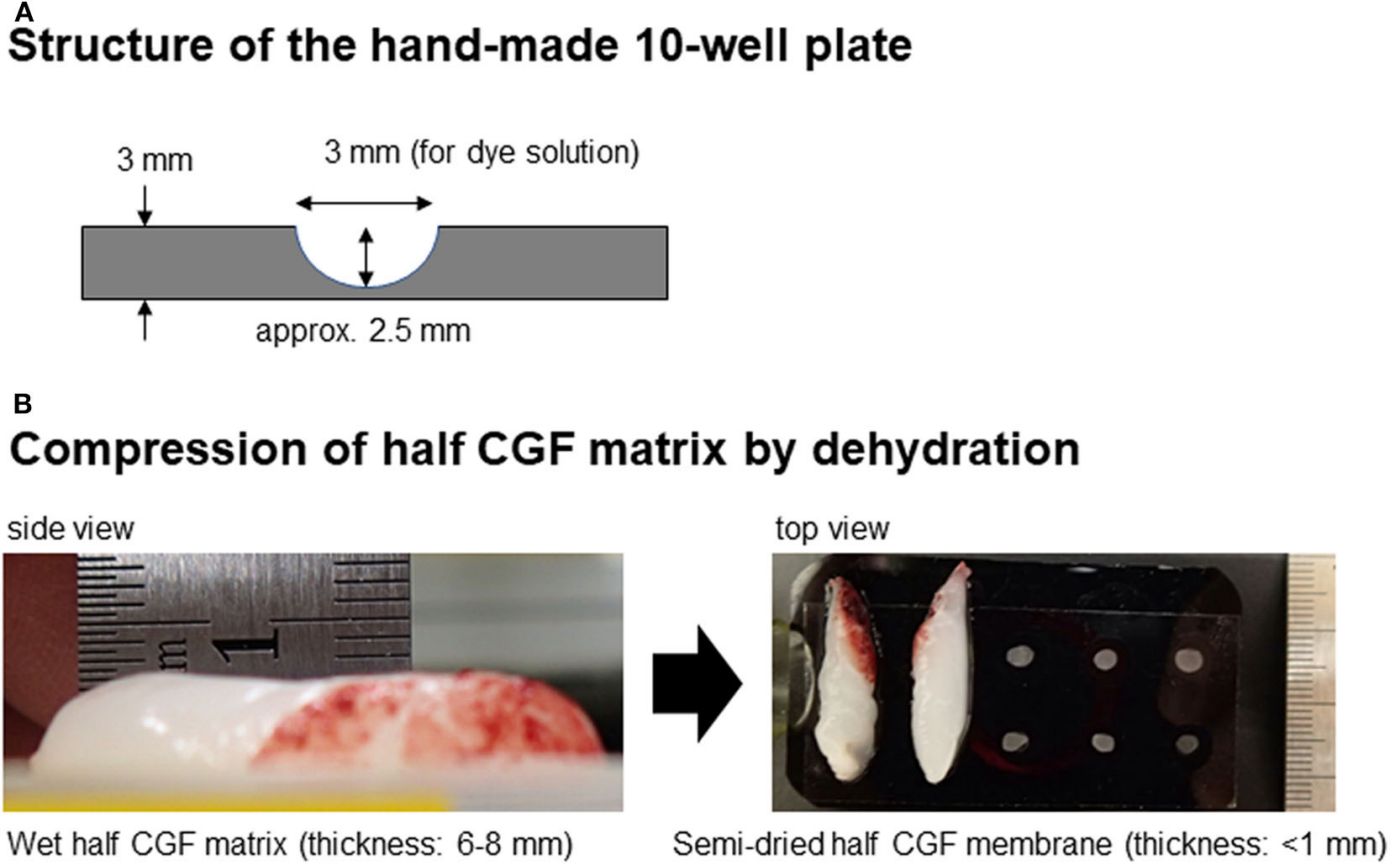
**(A)** Structure of the hand-made 10-well plate. **(B)** Appearance of wet CGF matrix and dehydrated membranous CGF matrix and positional relationship between the CGF membranes and wells.

The relationship between fluorescence intensity and NIR dye-conjugated secondary antibody was examined in the absence of platelets. The antibody solution was initially diluted at 1:10 in 0.1T-PBS and then serially diluted. Each dilution was transferred to a well of a hand-made black 10-well plate (Figure 2A). The plate was covered with a highly transparent seal and scanned using a Pearl Imaging system. The corresponding background value was subtracted from each measured value.

To evaluate the reduction in fluorescence intensity by dehydrated CGF membranes, the 10-well plates that were filled with diluted dye solutions and shielded were covered by CGF membranes (Figure 2B) and scanned as described above.

### Correlation Between Fluorescence Intensity and Suspended Platelets Count

PRP was prepared from whole-blood samples anticoagulated using acid-citrate-dextrose formula A (Terumo, Tokyo, Japan) by the double-centrifugation method (Aizawa et al., 2020). The platelets were suspended in PBS containing 0.1% bovine serum albumin and fixed in 20% neutralized formalin at a final concentration of 10% (FUJIFILM Wako Pure Chemical Co. Ltd.) for 30 min. The platelets were washed twice with 0.1T-PBS and probed with CD41 antibodies in BA-TPBS for 24 h. As in the case of CGF matrices, single platelets were washed and probed with secondary antibodies for 90 min. After washing, the platelets were suspended in 0.1T-PBS and scanned in a black 96-well plate (Nunc, Rochester, NY, USA). Platelet count was determined using an automated hematology analyzer.

### Correlation Between Fluorescence Intensity and Platelet Count in CGF Matrices

To measure the fluorescence intensity of known platelet counts in CGF matrices, platelet-poor plasma (PPP) and PRP were prepared in plain plastic tubes by the double-centrifugation method from whole blood samples in the absence of anticoagulants and immediately centrifuged again in glass tubes using a Medifuge centrifuge. The resulting platelet-poor and -rich fibrin matrices were quickly washed in PBS and fixed in 10% neutralized formalin for 3 h. The matrices were examined immunohistochemically and their fluorescence intensities were measured as described above.

### Statistical Analysis

**Figure 3:**
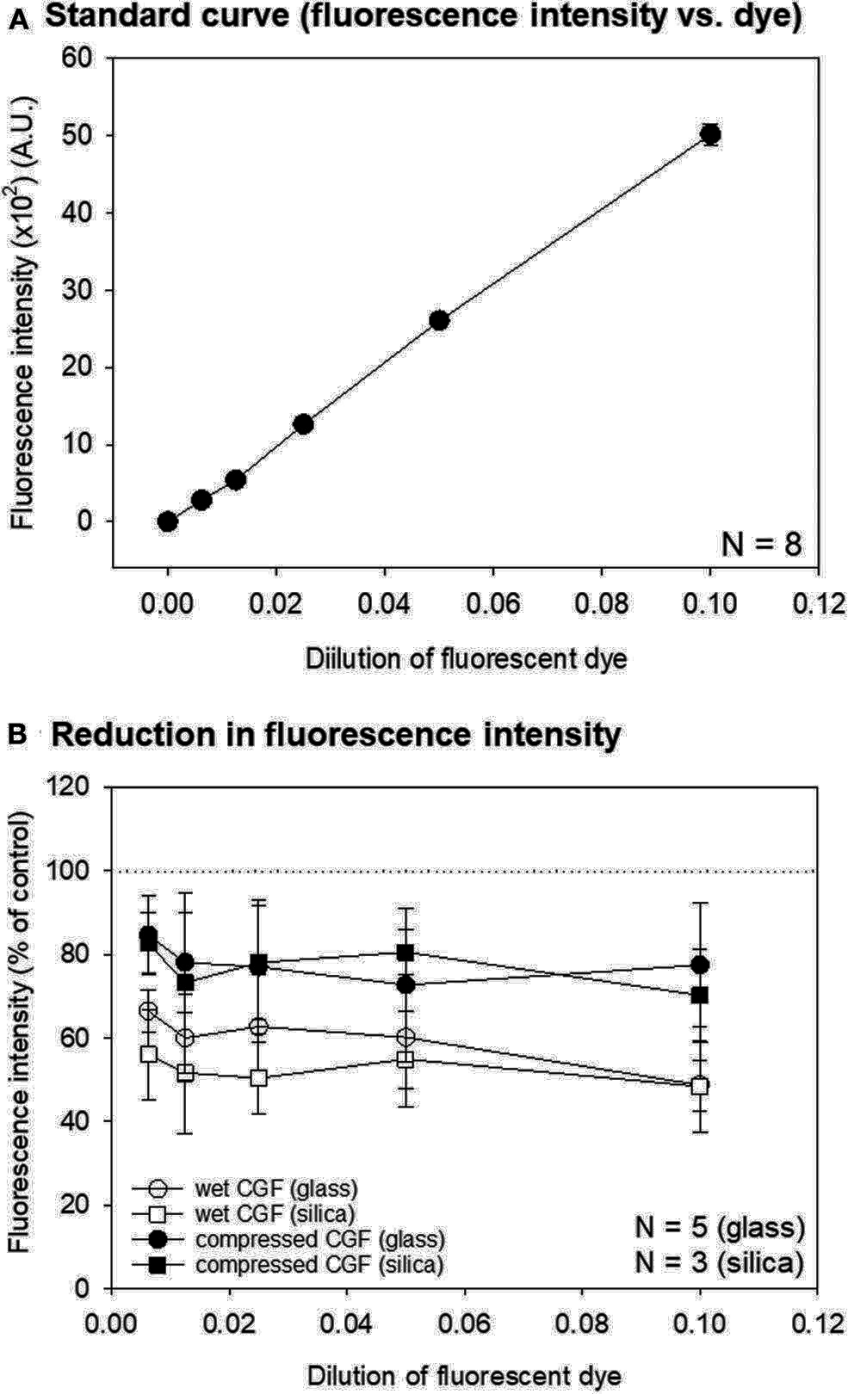
**(A)** A standard curve of the relationship between NIR dye dilution and fluorescence intensity. *N* = 8. **(B)** Reduction in the fluorescence intensity of wet and compressed CGF matrices prepared using glass and silica-coated plastic tubes on the standard curve. *N* = 5 (CGF membranes prepared using glass tubes), *N* = 3 (CGF membranes prepared using silica-coated tubes). No statistical difference was observed between glass and silica-coated tubes.

**Figure 4:**
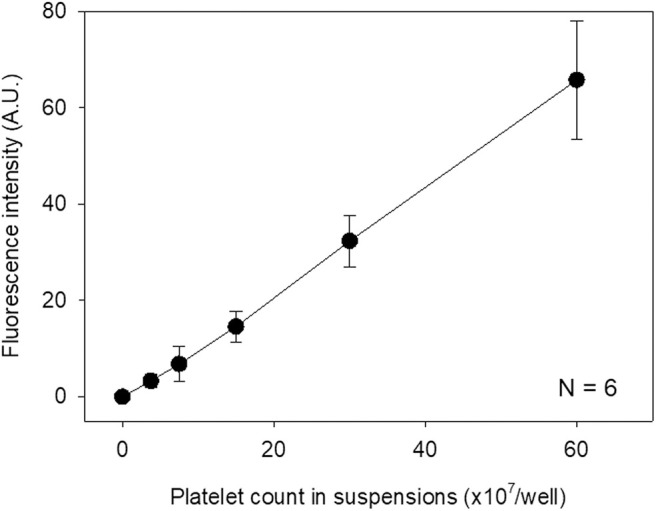
Standard curve for immunostained platelet count and fluorescence intensity. Immunostained platelets were suspended in 0.1T-PBS, counted, and scanned. *N* = 6.

**Figure 5:**
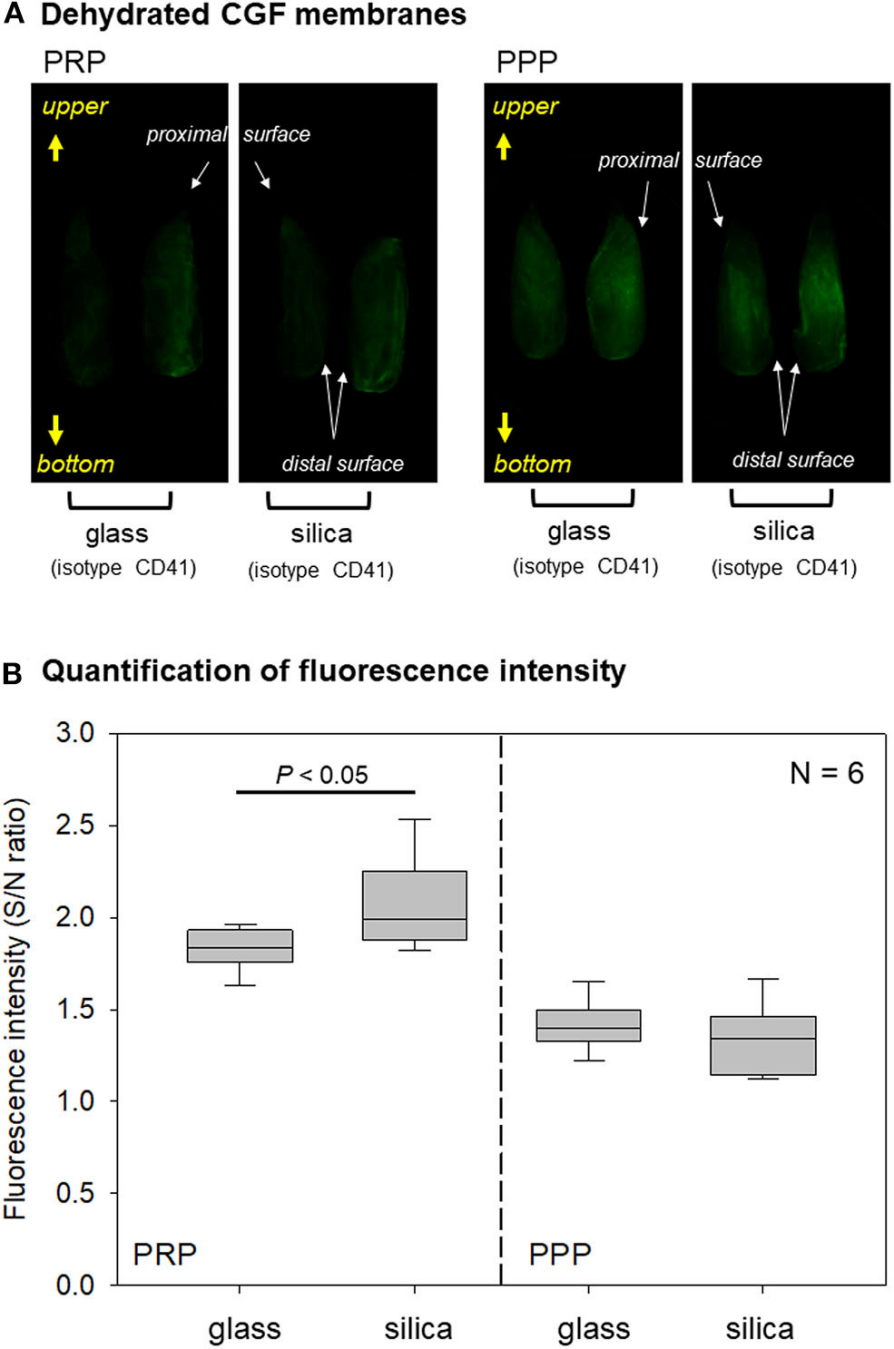
**(A)** Macroscopic observations of immunostained PPP- and PRP-derived fibrin membranes. A couple of half fibrin matrices, which were derived from the same fibrin matrices, were used for non-specific detection using an isotype control (left) and specific detection using a CD41 antibody (right). These observations are representative of the other five samples obtained from different donors. **(B)** Box plot of fluorescence intensity in PPP- and PRP-derived fibrin matrices prepared using glass and silica-coated plastic tubes *N* = 6.

The data are expressed as mean ± standard deviation in Figures 3, 4 or represented by box plots in Figures 5, **7**. For two-group comparisons, Student's *t*-test was performed to compare mean values (SigmaPlot 12.5; Systat Software, Inc., San Jose, CA, USA). *P* < 0.05 was considered statistically significant. Linear regression was carried out and the coefficient of determination (*R*^2^) was calculated by SigmaPlot.

## Results

### Correlation Between Measured Fluorescence Intensity and Fluorescence Dye Dilution or Immunostained Suspended Platelets

To validate the accuracy of quantification based on analysis of the NIR imaging system, we examined the relationship between measured fluorescence intensity and fluorescence dye dilution. As shown in Figure 3A, these parameters exhibited a linear relationship within a wide range. Possible inhibitory effects of wet CGF matrices or dehydrated CGF membranes on fluorescence dye intensity were then examined. As shown in Figure 3B, regardless of tube type, wet CGF matrices (6–8 mm in thickness) reduced the signal intensity by ~40–50%, while dehydrated CGF membranes (~1 mm in thickness) reduced the signal by ~20%.

The relationship between the measured fluorescence intensity of immunostained platelets and platelet count in suspension was examined. As revealed in Figure 4, a linear standard curve was obtained between these parameters within a wide range as observed above.

### Correlation Between Fluorescence Intensity and Immuno-Stained Platelets Contained in CGF Matrices

The quality of the data obtained was sufficient for validating the accuracy of the quantification of the imaging system. However, fixed fibrin fibers possibly influence the quantification by scattering, reflection, and shielding. Thus, we examined the relationship between the measured fluorescence intensity of immunostained fibrin matrices and platelet count in the fibrin matrices. Because it takes ~24 h to determine platelet count in fibrin matrices using the digestion method (Kitamura et al., 2018b), we first prepared PPP and PRP and counted platelets before centrifugation.

Imaging data of platelet distribution in dehydrated CGF membranes are shown in Figure 5A. In PRP-derived fibrin membranes, regardless of tube type, CD41-specific signals were more apparent than non-specific signals. By contrast, in PPP-derived fibrin membranes, the fluorescence signals were similar. As shown in Figure 5B, quantification of these data clarified that higher fluorescence intensities, expressed by the signal-to-noise ratio, were obtained in PRP-derived fibrin membranes compared with those in PPP-derived fibrin membranes. In addition, a significant difference was observed between glass and silica-coated plastic tubes only in PRP-derived fibrin membranes.

**Figure 6:**
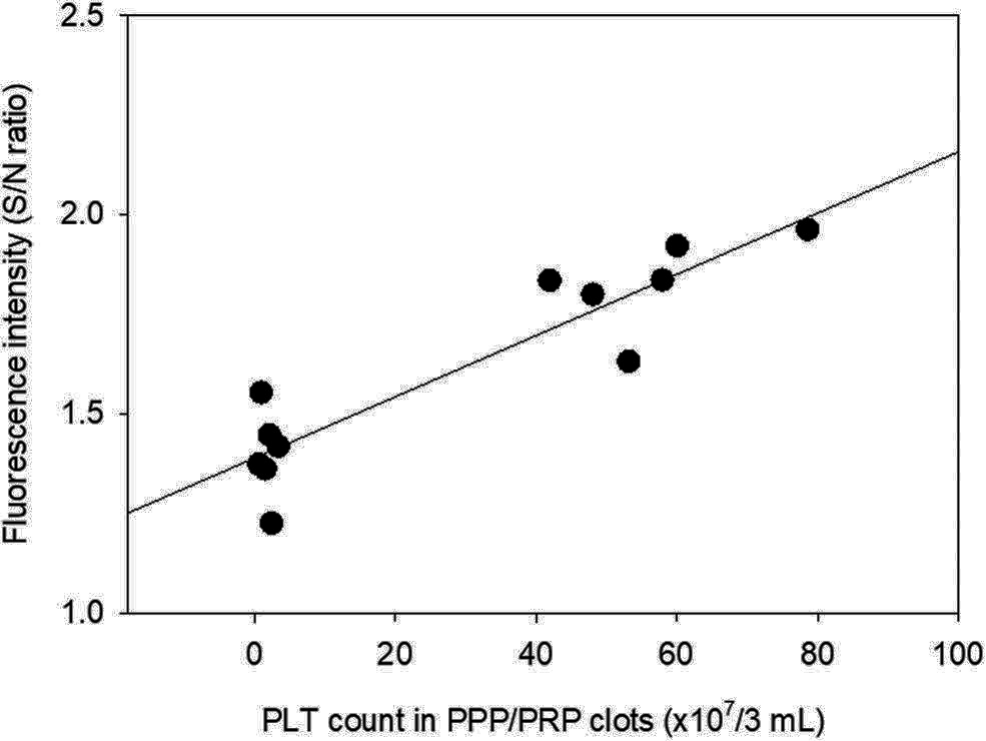
Scatter plot of the relationship between platelet count in PPP and PRP preparations used for fibrin matrix formation and fluorescence intensity in the resulting PPP- and PRP-derived fibrin matrices. Platelet count was determined in the absence of anticoagulants prior to preparation of fibrin matrices *N* = 6.

The relationship between measured fluorescence intensity and platelet count in dehydrated fibrin membranes prepared using glass tubes is represented by a scatter plot in Figure 6. A strong correlation was observed between fluorescence intensity and platelet count in dehydrated fibrin membranes (*R*^2^ = 0.838).

### Examination of CGF Matrices Prepared Using Plain Glass Tubes and Silica-Coated Plastic Tubes

**Figure 7:**
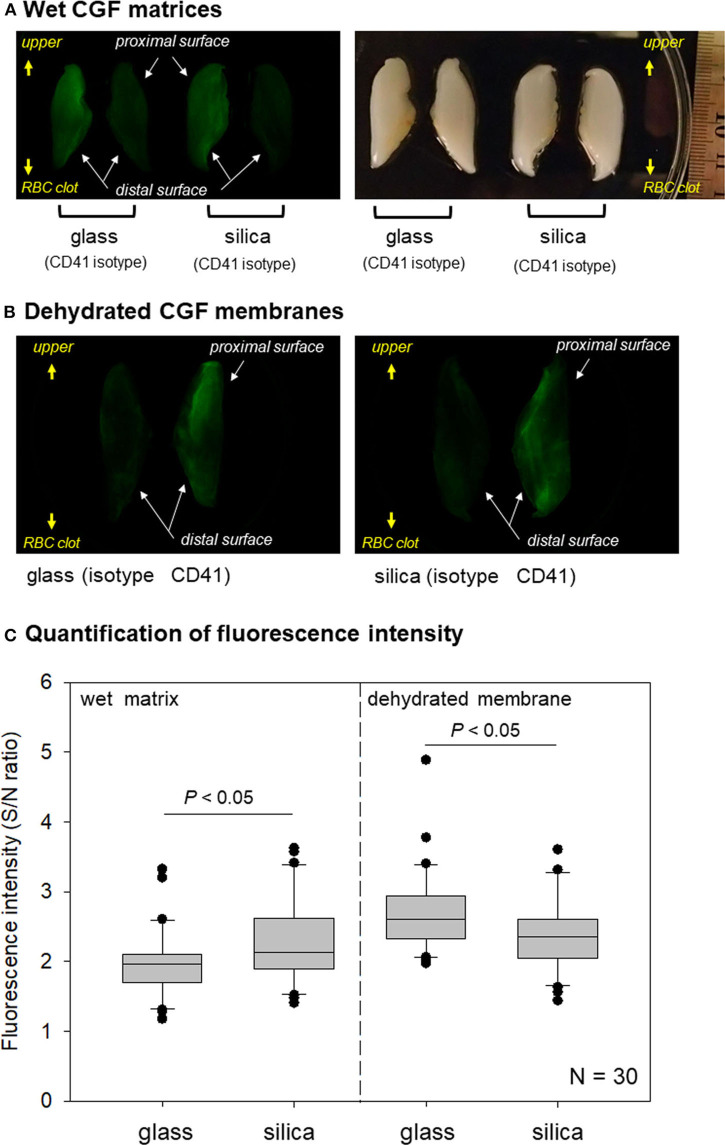
**(A)** Macroscopic observations of immunostained wet CGF matrices. A couple of half fibrin matrices, which were derived from the same fibrin matrices, were used for non-specific detection using an isotype control (right) and specific detection using a CD41 antibody (left). These observations are representative of the other five samples obtained from different donors. **(B)** Macroscopic observations of immunostained dehydrated CGF membranes. A couple of half fibrin membranes, which were derived from the same fibrin matrices, were used for non-specific detection using an isotype control (left) and specific detection using a CD41 antibody (right). These observations are representative of the other five samples obtained from different donors. **(C)** Box plot of fluorescence intensity in wet and dehydrated membranous CGF matrices prepared using glass and silica-coated plastic tubes *N* = 6.

Through these preliminary investigations, we validated that this method was applicable for evaluating platelet distribution in CGF matrices. Images of platelet distribution in wet and dehydrated membranous CGF matrices are shown in Figures 7A,B. In both images, glass tubes gathered platelets at distal surface regions, while silica-coated tubes distributed platelets dispersively but homogeneously. However, clearer images were obtained in dehydrated CGF membranes.

Total fluorescence intensity was measured and expressed as signal-to-noise ratio in Figure 7C. As observed in dehydrated PRP-derived fibrin matrices (Figure 5B), the specific fluorescence signals were significantly higher in wet silica-prepared CGF matrices than in wet glass-prepared ones. By contrast, in dehydrated samples, the specific fluorescence signals in glass-prepared matrices was higher than those in silica-prepared matrices.

**Figure 8:**
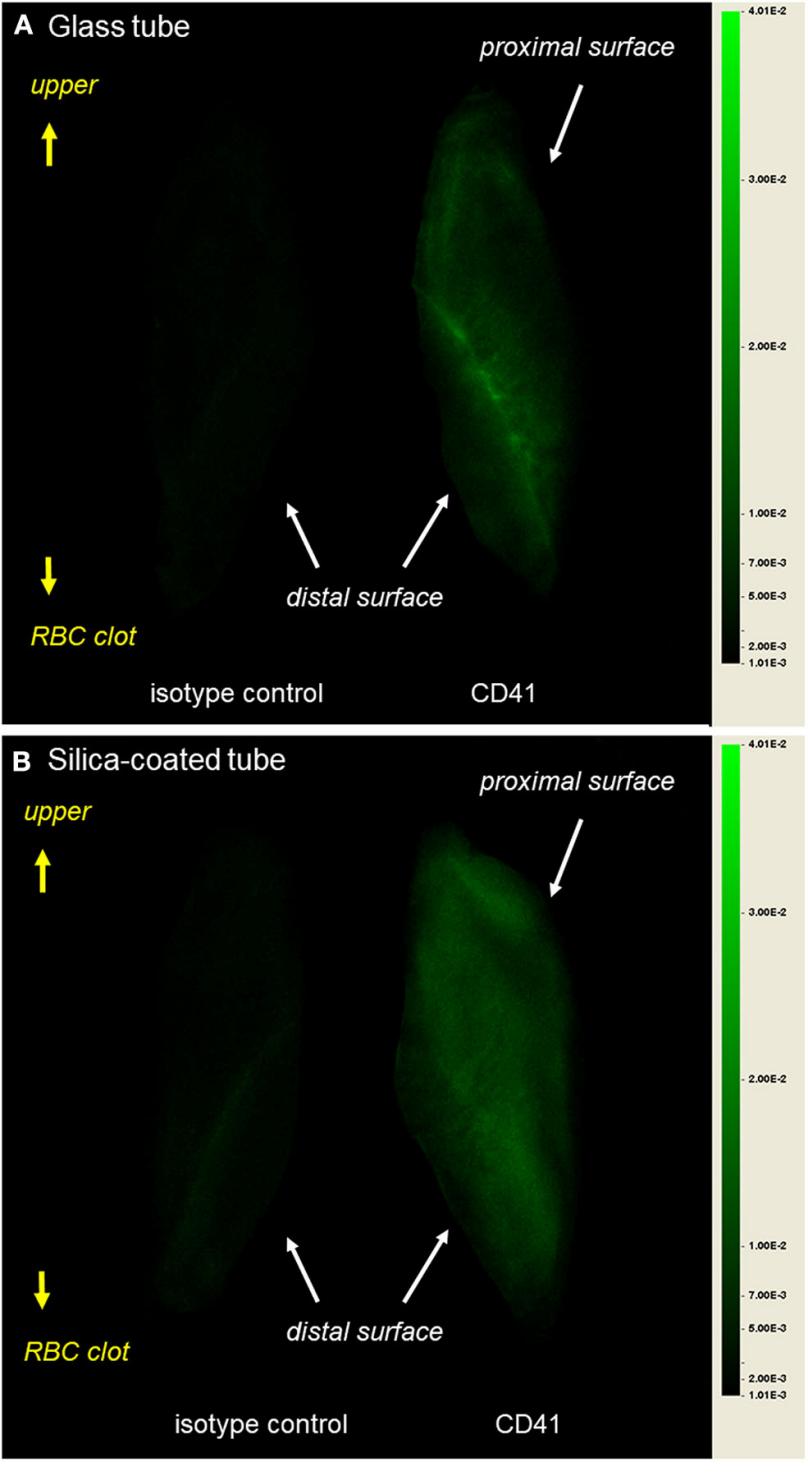
Digitally enlarged macroscopic observations of immunostained dehydrated CGF membranes prepared using **(A)** glass tube and **(B)** silica-coated plastic tubes. These observations are representative of the other five samples obtained from different donors.

To examine platelet distribution in dehydrated CGF membranes more carefully, images were randomly selected from different sets of samples and digitally enlarged, as shown in Figure 8. Although the platelets were relatively gathered at the outer edge of the interface to the RBC clot in the glass-prepared CGF membrane, other platelets were generally distributed in the distal surface region. By contrast, platelets were distributed homogeneously in the silica-prepared CGF membrane.

## Discussion

### Difference in Platelet Distribution in Glass- and Silica-Prepared CGF Matrices

From the results of SEM and additionally of immunohistochemical staining or simulation studies using liquid PRF preparations (Kobayashi et al., 2012; Watanabe et al., 2017; Miron et al., 2019a; Tsujino et al., 2019b), it is generally believed that centrifugation distributes platelets based on the difference in density gradient in the PRF matrix and in the plasma fraction of citrated blood samples. Thus, platelet density decreases as the distance to the rotor axis decreases (Kobayashi et al., 2012; Ghanaati et al., 2014; Dohan Ehrenfest et al., 2018; El Bagdadi et al., 2019; Miron et al., 2019a). However, these modalities are limited in that such morphological data are semi-quantitative and at times inappropriately represent the corresponding data because of technical or operational bias. To overcome this shortcoming, it is necessary to evaluate individual PRF matrices from the surface to deep regions without sectioning. In this study, we applied an NIR imaging technology to examine whole-fibrin matrices and successfully demonstrated platelet distribution in the CGF matrices (for technical details, see the following subsections).

The main finding of this study is that platelet distribution in glass-prepared CGF matrices is distinguishable from that in silica-prepared ones. In the glass-prepared CGF matrices, platelets were distributed mainly on the distal side of the surface regions. In addition, platelets did not necessarily decrease in number with increasing distance from the interface of the red thrombus and still existed on the upper regions in all samples tested. By contrast, platelets were distributed widely and homogeneously in the silica-prepared CGF matrices. In a previous study of CGF matrices (Kobayashi et al., 2012), we examined platelet distribution on the surface regions using SEM and obtained similar findings. While comparing glass tubes to silica-coated tubes (Tsujino et al., 2019b), we previously demonstrated that high-speed centrifugation accumulated platelets on the distal surface regions in glass-prepared matrices, but homogeneously in silica-prepared matrices.

In addition, although it is well-known that individual variations are generally large in terms of the characteristics of platelet concentrates, this study demonstrated that platelet distribution in CGF (and probably other platelet-concentrated fibrin) matrices can be classified in pattern by the type of blood collection tube.

### What Causes the Difference in Platelet Distribution Pattern?

**Figure 9:**
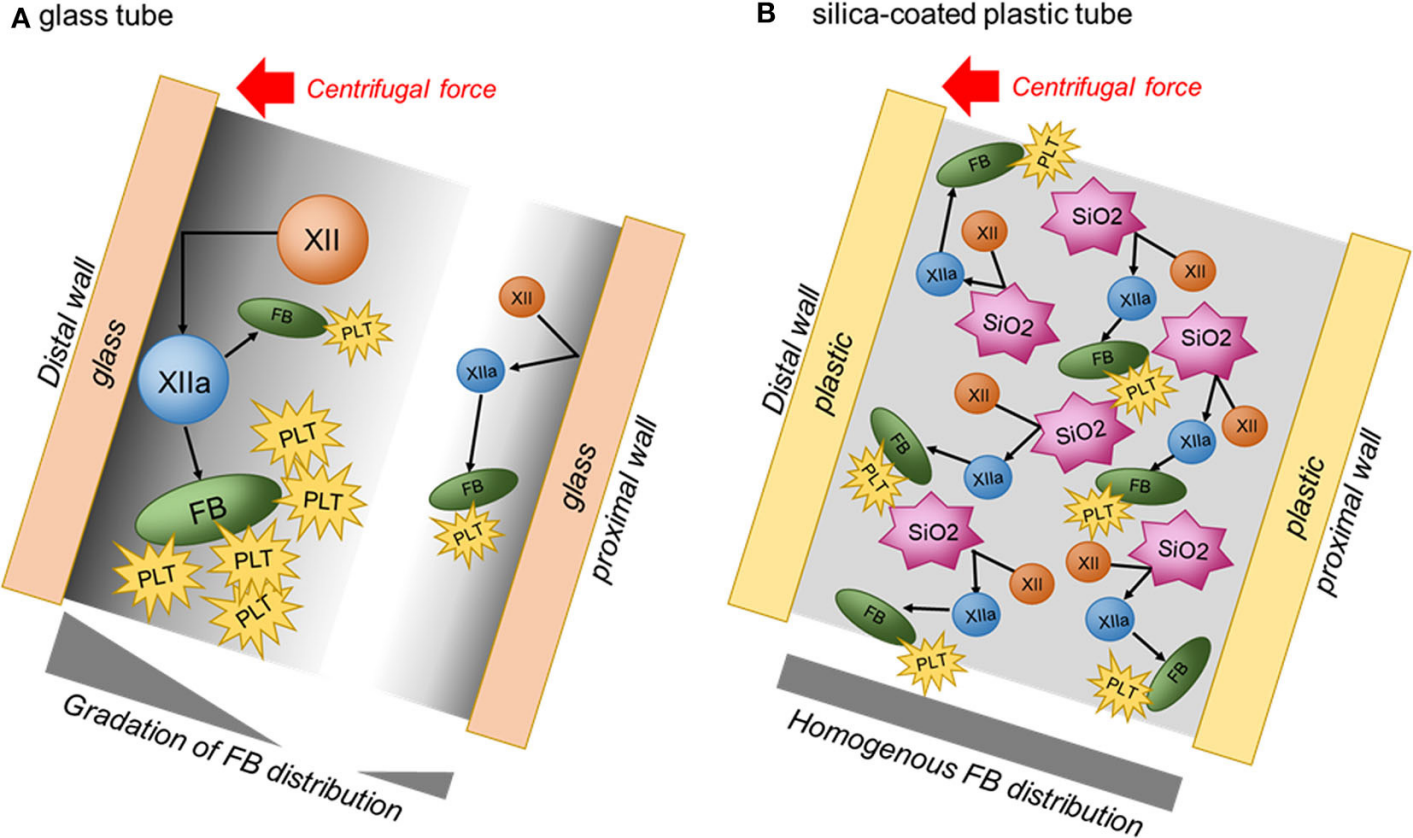
Illustrations of possible difference in coagulation mechanisms in plain glass and silica-coated tubes. **(A)** Glass tube. **(B)** Silica-coated plastic tube. FB, fibrin; PLT, platelet; XII, coagulation factor XII; XIIa, activated coagulation factor XII; SiO2, silica microparticle.

With low-speed centrifugation, it is difficult to observe a difference in platelet distribution. However, as the centrifugation speed increases, platelets are gradually accumulated on the distal side of glass tubes and thus the difference in platelet distribution is easily recognized in comparison to that in silica-coated tubes (Tsujino et al., 2019b). This difference can be explained by the different mechanisms of matrix formation (Figure 9). In glass tubes, blood cells and relatively large proteins contained in plasma, such as coagulation factor XII (Mr = 80 kDa), are thought to be pressed on the distal wall by centrifugal force to more easily and efficiently activate the intrinsic coagulation cascade. Thus, it is speculated that initial contact takes place more efficiently on the distal surface of the tube. As fibrin fibers, which are end products of the coagulation cascade, are formed more actively, platelets are expected to aggregate on fibrin fibers more frequently. Coagulation could be induced even on the proximal wall, but its activity is thought to be much lower than on the distal wall. As a result, fibrin fibers and platelets are preferentially distributed on the distal side of CGF matrices.

On the contrary, in silica-coated plastic tubes, silica microparticles are easily and immediately detached from the inside wall and suspended in blood samples as blood is aspirated into the tubes. Silica microparticles existing ubiquitously in blood samples efficiently activate the coagulation reaction and thus form fibrin fibers homogeneously in the CGF matrix. This speculation is supported by the observation that silica-prepared matrices usually possess higher water-holding capacity, as evaluated by the thickness of the half-matrix (Figure 2), than that of glass-prepared matrices.

### Limitations of this Imaging Technique

Conventional SEM can be used to examine platelet distribution on the surface regions of platelet-concentrated fibrin matrices. However, observations representative of other hidden accumulated data are sometimes biased by the most frequently observed findings and/or investigators with strongly “fixed” hypotheses. On the contrary, conventional immunohistochemical methods only provide information on selected two-dimensional planes (i.e., sections) of samples. Three-dimensional (3D) image reconstruction theoretically makes it possible to understand more precisely how platelets are distributed in the matrix at higher magnifications from serial sections. However, this process is complicated, time-consuming, and laborious, and immunohistochemical data are not fully quantitative at similar staining levels.

Even though long incubation time is required, the NIR imaging method conveniently provides quantitative data on platelet distribution by the principle behind projection of three-dimensional objects to two-dimensional planes. Moreover, the NIR imager used in this study has a wide dynamic range and can therefore sensitively detect and quantify targeted markers. Although there are considerable technical limitations (see below), we suggest that the data obtained are reliable not only qualitatively but also quantitatively.

In terms of technical limitations, it should be noted that PRF matrices are composed of intertwined and cross-linked fine fibrin fibers. Thus, in addition to attenuation by the “inverse-square law,” the fluorescence signal is scattered, absorbed, reflected, shielded, and diffused so that its intensity is generally attenuated and the image becomes blurred. In this study, we observed that the images of platelet distribution in wet CGF matrices looked slightly hazy and that the fluorescence intensity of wet CGF matrices was lower than that of dehydrated CGF membranes.

As for the shielding effects of dehydrated CGF membranes, fluorescence intensity was reduced by ~20% at 800 nm. Although these effects may negatively influence the quantitative capability, the simultaneous acquisition of both quantitative and qualitative data is the biggest advantage of this technique.

### Clinical Relevance

Because silica (SiO_2_) is a component of glass, some researchers believe that fibrin matrices with almost equal quality can be prepared using silica-coated tubes. In fact, silica-coated tubes have been recommended for PRF preparation (Dohan et al., 2006; O'Connell, 2007; Dohan Ehrenfest et al., 2012), but the reported results vary. In the slow-speed centrifugation for advanced PRF (A-PRF) preparation, using the immunohistochemical method, we previously demonstrated that platelet distribution in silica-prepared matrices was distinguishable from that in glass-prepared ones (Tsujino et al., 2019b). Similarly, adopting high-speed centrifugation in this study, platelet distribution was observed to be different. Taken together with our previous findings regarding silica contamination and cytotoxicity (Tsujino et al., 2019b,c; Masuki et al., 2020) these data suggest that silica-coated tubes cannot be used to prepare any type of PRF matrix with almost equal quality as that of glass tubes.

At present, the type of NIR imaging device used in this study is expensive (>100,000 USD), and the proposed method requires 2–3 days of preparation. Thus, even though the running cost is actually low, it is difficult to standardize or popularize this method for routine point-of-care testing. However, this is a promising technology to ensure the quality of platelet-concentrated fibrin matrices for more reliable and highly qualified randomized clinical trials (Kitamura et al., 2018a; Kawase et al., 2019; Tsujino et al., 2019a).

We have claimed the importance and necessity of quality assurance for any type of platelet concentrate used in regenerative therapy (Kawase et al., 2019). Standardization of preparation protocol is also important but not perfect for standardization of product and therapeutic quality (Kobayashi et al., 2012; Gomez et al., 2015; Chahla et al., 2017; Fadadu et al., 2019; Miron et al., 2019a,b; Saltzman et al., 2020). To minimize the difference in clinical outcomes by excluding outliers (e.g., fewer platelet-concentrated samples) and improve therapy to become more predictable, individual products should be tested based on standardized criteria. In the case of anticoagulated liquid forms of PRP and its derivatives, the quantity and quality of platelets and white blood cells can be evaluated and considered the main criteria (Kitamura et al., 2018a; Tsujino et al., 2019a; Aizawa et al., 2020). The coagulation activity and platelet activation status may be counted as additional criteria (Milants et al., 2017). By contrast, in the case of solid forms of PRF and its derivatives, platelet count and activity can hardly be evaluated for technical limitations, even though the coagulation activity may be evaluated by the appearance or strength of the resulting fibrin fibers (Isobe et al., 2017a,b; Kawabata et al., 2017).

Our novel imaging method enabled us to evaluate platelet count and distribution in insoluble fibrin matrices. We previously developed the digestion method to accurately determine platelet count (Kitamura et al., 2018b). However, this method did not reveal the exact part of the platelet-concentrated fibrin matrix without careful dissection. Instead, the current study clarified that platelets are not concentrated in the bottom region, which corresponds to the “buffy coat” in PRP preparation. Thus, our previous (Tsujino et al., 2019b) and present data cooperatively caution against the selective collection of “buffy coat” regions, which has often been performed for better clinical outcome, especially by “well-informed” clinicians. From a physical and medical point of view, it would be better to use whole PRF matrices at once than to examine individual divided regions and subsequently combine the data.

## Conclusions

The imaging technique that we developed and introduced in this study enables to evaluate platelet quantity and distribution at once in insoluble fibrin matrices. We believe that this technique will contribute to standardizing the quality of platelet-concentrated fibrin matrices for regenerative therapy. Using this technique, we demonstrated that regardless of centrifugal speed, PRF matrices prepared using silica-coated tubes are distinguishable from those prepared using glass tubes.

## Data Availability Statement

The raw data supporting the conclusions of this article will be made available by the authors, without undue reservation, to any qualified researcher.

## Ethics Statement

The studies involving human participants were reviewed and approved by the Ethics Committee for Human Subjects of the Niigata University School of Medicine (Niigata, Japan). The patients/participants provided their written informed consent to participate in this study. Written informed consent was obtained from the individual(s) for the publication of any potentially identifiable images or data included in this article.

## Author Contributions

SY and TK: conceptualization. TK: methodology, writing—review and editing, and funding acquisition. HA and TT: validation. AS: formal analysis. SY, HA, AS, TT, KI, TW, YK, and TK: investigation. KI and TW: data curation. HA, TT, CM, and TK: writing—original draft preparation. CM and TK: writing—review and editing. TT: visualization. HO: supervision. TW: project administration. All authors have read and agreed to the published version of the manuscript.

## Conflict of Interest

The authors declare that the research was conducted in the absence of any commercial or financial relationships that could be construed as a potential conflict of interest.

## References

[B1] Aizawa H. Kawabata H. Sato A. Masuki H. Watanabe T. Tsujino T.. (2020). A comparative study of the effects of anticoagulants on pure platelet-rich plasma quality and potency. Biomedicines 8:42. 10.3390/biomedicines8030042 32106422 PMC7148468

[B2] Chahla J. Cinque M. E. Piuzzi N. S. Mannava S. Geeslin A. G. Murray I. R.. (2017). A call for standardization in platelet-rich plasma preparation protocols and composition reporting: a systematic review of the clinical orthopaedic literature. J. Bone Joint Surg. 99, 1769–1779. 10.2106/JBJS.16.01374 29040132

[B3] Dohan Ehrenfest D. M. Bielecki T. Jimbo R. Barbe G. Del Corso M. Inchingolo F.. (2012). Do the fibrin architecture and leukocyte content influence the growth factor release of platelet concentrates? An evidence-based answer comparing a pure platelet-rich plasma (P-PRP) gel and a leukocyte- and platelet-rich fibrin (L-PRF). Curr. Pharm. Biotechnol. 13, 1145–1152. 10.2174/138920112800624382 21740377

[B4] Dohan Ehrenfest D. M. Pinto N. R. Pereda A. Jimenez P. Corso M. D. Kang B. S.. (2018). The impact of the centrifuge characteristics and centrifugation protocols on the cells, growth factors, and fibrin architecture of a leukocyte- and platelet-rich fibrin (L-PRF) clot and membrane. Platelets 29, 171–184. 10.1080/09537104.2017.1293812 28437133

[B5] Dohan D. M. Choukroun J. Diss A. Dohan S. L. Dohan A. J. Mouhyi J.. (2006). Platelet-rich fibrin (PRF): a second-generation platelet concentrate. Part I: technological concepts and evolution. Oral Surg. Oral Med. Oral Pathol. Oral Radiol. Endod. 101, e37–44. 10.1016/j.tripleo.2005.07.008 16504849

[B6] El Bagdadi K. Kubesch A. Yu X. Al-Maawi S. Orlowska A. Dias A.. (2019). Reduction of relative centrifugal forces increases growth factor release within solid platelet-rich-fibrin (PRF)-based matrices: a proof of concept of LSCC (low speed centrifugation concept). Eur. J Trauma Emerg. Surg. 45, 467–479. 10.1007/s00068-017-0785-7 28324162 PMC6579868

[B7] Fadadu P. P. Mazzola A. J. Hunter C. W. Davis T. T. (2019). Review of concentration yields in commercially available platelet-rich plasma (PRP) systems: a call for PRP standardization. Reg. Anesth. Pain Med. 16:rapm-2018–100356. 10.1136/rapm-2018-100356 30992411

[B8] Ghanaati S. Booms P. Orlowska A. Kubesch A. Lorenz J. Rutkowski J.. (2014). Advanced Platelet-Rich Fibrin (A-PRF) - A new concept for cell-based tissue engineering by means of inflammatory cells. J. Oral Implantol. 40, 679–89. 10.1563/aaid-joi-D-14-00138 24945603

[B9] Gomez L. A. Escobar M. Penuela O. (2015). Standardization of a protocol for obtaining platelet rich plasma from blood donors; a tool for tissue regeneration procedures. Clin. Lab. 61, 973–980. 10.7754/Clin.Lab.2015.141141 26427141

[B10] Isobe K. Suzuki M. Watanabe T. Kitamura Y. Suzuki T. Kawabata H.. (2017a). Platelet-rich fibrin prepared from stored whole-blood samples. Int. J. Implant. Dent. 3:6. 10.1186/s40729-017-0068-4 28251561 PMC5332319

[B11] Isobe K. Watanebe T. Kawabata H. Kitamura Y. Okudera T. Okudera H.. (2017b). Mechanical and degradation properties of advanced platelet-rich fibrin (A-PRF), concentrated growth factors (CGF), and platelet-poor plasma-derived fibrin (PPTF). Int. J. Implant. Dent. 3:17. 10.1186/s40729-017-0081-7 28466249 PMC5413460

[B12] Kawabata H. Isobe K. Watanabe T. Okudera T. Nakamura M. Suzuki M.. (2017). Quality assessment of platelet-rich fibrin-like matrix prepared from whole blood samples after extended storage. Biomedicines 5:57. 10.3390/biomedicines5030057 28926988 PMC5618315

[B13] Kawase T. (2015). Platelet-rich plasma and its derivatives as promising bioactive materials for regenerative medicine: basic principles and concepts underlying recent advances. Odontology 103, 126–135. 10.1007/s10266-015-0209-2 26040505

[B14] Kawase T. Takahashi A. Watanabe T. Tsujino T. (2019). Proposal for point-of-care testing of platelet-rich plasma quality. Int. J. Growth Factors Stem Cells Dent. 2, 13–17. 10.4103/GFSC.GFSC_26_18

[B15] Kawase T. Tanaka T. (2017). An updated proposal for terminology and classification of platelet-rich fibrin. Regen. Ther. 7, 80–81. 10.1016/j.reth.2017.10.002 30271855 PMC6153447

[B16] Kitamura Y. Suzuki M. Tsukioka T. Isobe K. Tsujino T. Watanabe T.. (2018a). Spectrophotometric determination of platelet counts in platelet-rich plasma. Int. J. Implant. Dent. 4:29. 10.1186/s40729-018-0140-8 30276491 PMC6167270

[B17] Kitamura Y. Watanabe T. Nakamura M. Isobe K. Kawabata H. Uematsu K.. (2018b). Platelet counts in insoluble platelet-rich fibrin clots: a direct method for accurate determination. Front. Bioeng. Biotechnol. 6:4. 10.3389/fbioe.2018.00004 29450197 PMC5799223

[B18] Kobayashi M. Kawase T. Horimizu M. Okuda K. Wolff L. F. Yoshie H. (2012). A proposed protocol for the standardized preparation of PRF membranes for clinical use. Biologicals 40, 323–329. 10.1016/j.biologicals.2012.07.004 22841724

[B19] Marx R. E. Carlson E. R. Eichstaedt R. M. Schimmele S. R. Strauss J. E. Georgeff K. R. (1998). Platelet-rich plasma: growth factor enhancement for bone grafts. Oral Surg. Oral Med. Oral Pathol. Oral Radiol. Endod. 85, 638–646. 10.1016/S1079-2104(98)90029-4 9638695

[B20] Masuki H. Isobe K. Kawabata H. Tsujino T. Yamaguchi S. Watanabe T.. (2020). Acute cytotoxic effects of silica microparticles used for coating of plastic blood-collection tubes on human periosteal cells. Odontology. 10.1007/s10266-020-00486-z. [Epub ahead of print]. 31997225 PMC7438384

[B21] Milants C. Bruyere O. Kaux J. F. (2017). Responders to platelet-rich plasma in osteoarthritis: a technical analysis. BioMed Res. Int. 2017:7538604. 10.1155/2017/7538604 28904970 PMC5585615

[B22] Miron R. J. Chai J. Zhang P. Li Y. Wang Y. Mourao C.. (2019a). A novel method for harvesting concentrated platelet-rich fibrin (C-PRF) with a 10-fold increase in platelet and leukocyte yields. Clin. Oral Invest. 10.1007/s00784-019-03147-w [Epub ahead of print]. 31788748

[B23] Miron R. J. Pinto N. R. Quirynen M. Ghanaati S. (2019b). Standardization of relative centrifugal forces in studies related to platelet-rich fibrin. J. Periodontol. 90, 817–820. 10.1002/JPER.18-0553 30730050

[B24] O'Connell S. M. (2007). Safety issues associated with platelet-rich fibrin method. Oral Surg. Oral Med. Oral Pathol. Oral Radiol. Endod. 103, 587–593. 10.1016/j.tripleo.2007.03.017 17466883

[B25] Saltzman B. M. Frank R. M. Davey A. Cotter E. J. Redondo M. L. Naveen N.. (2020). Lack of standardization among clinical trials of injection therapies for knee osteoarthritis: a systematic review. Phys. Sportsmed. 26, 1–24. 10.1080/00913847.2020.1726716 32027200

[B26] Smith A. M. Mancini M. C. Nie S. (2009). Bioimaging: second window for *in vivo* imaging. Nat Nanotechnol 4, 710–711. 10.1038/nnano.2009.326 19898521 PMC2862008

[B27] Tsujino T. Isobe K. Kawabata H. Aizawa H. Yamaguchi S. Kitamura Y.. (2019a). Spectrophotometric determination of the aggregation activity of platelets in platelet-rich plasma for better quality control. Dent. J. 7:61. 10.3390/dj7020061 31163628 PMC6631196

[B28] Tsujino T. Masuki H. Nakamura M. Isobe K. Kawabata H. Aizawa H.. (2019b). Striking differences in platelet distribution between advanced-platelet-rich fibrin and concentrated growth factors: effects of silica-containing plastic tubes. J. Func. Biomater. 10:43. 10.3390/jfb10030043 31533279 PMC6787607

[B29] Tsujino T. Takahashi A. Yamaguchi S. Watanabe T. Isobe K. Kitamura Y.. (2019c). Evidence for contamination of silica microparticles in advanced platelet-rich fibrin matrices prepared using silica-coated plastic tubes. Biomedicines 7:45. 10.3390/biomedicines7020045 31248187 PMC6631693

[B30] Tsukioka T. Hiratsuka T. Nakamura M. Watanabe T. Kitamura Y. Isobe K.. (2019). An on-site preparable, novel bone-grafting complex consisting of human platelet-rich fibrin and porous particles made of a recombinant collagen-like protein. J. Biomed. Mater. Res. Part B Appl. Biomater. 107, 1420–1430. 10.1002/jbm.b.34234 30270545 PMC6585782

[B31] Watanabe T. Isobe K. Suzuki T. Kawabata H. Nakamura M. Tsukioka T.. (2017). An evaluation of the accuracy of the subtraction method used for determining platelet counts in advanced platelet-rich fibrin and concentrated growth factor preparations. Dent. J. 5:7. 10.3390/dj5010007 29563413 PMC5806990

